# Sporadic Appetites: Unearthing the Fungal Diets of Two Mycophagous Mammals Across an Australian Climate Gradient

**DOI:** 10.1002/ece3.73839

**Published:** 2026-06-11

**Authors:** Rebecca J. Quah, Saul J. Cowen, Robert A. Davis, Harriet R. Mills, Anna J. M. Hopkins

**Affiliations:** ^1^ Conservation and Biodiversity Research Centre, School of Science Edith Cowan University Joondalup Western Australia Australia; ^2^ Biodiversity and Conservation Science, Department of Biodiversity Conservation and Attractions Kensington Western Australia Australia; ^3^ School of Biological Sciences The University of Western Australia Crawley Western Australia Australia; ^4^ Perth Zoo Science, Department of Biodiversity Conservation and Attractions South Perth WA Australia

**Keywords:** conservation translocations, dietary resources, environmental DNA, Potoroidae

## Abstract

Translocating threatened ecosystem engineers may offer the dual benefit of contributing to species conservation and ecosystem restoration. Boodies (burrowing bettong; *Bettongia lesueur* ) and woylies (brush‐tailed bettong; *Bettongia ogilbyi*) are threatened mycophagous marsupials known for their crucial role in ecosystem health through fungal dispersal and bioturbation. As part of restoration efforts across Australia, these bettong species are frequently targeted for translocations, where a better understanding of their fungal dietary requirements is essential for ensuring their resource requirements are met and in predicting their ecological contributions to recipient ecosystems. Here, we used DNA metabarcoding to study the fungal diets of boodies and woylies from 90 scat samples collected across five sites spanning Mediterranean‐type to arid climates. DNA metabarcoding identified 379 fungal amplicon sequence variants (ASVs) from scat samples, of which 82 ASVs were identified as macrofungi, likely to be deliberately consumed by boodies and woylies. We found that both species of bettongs demonstrated similar fungal dietary diversity profiles, with fungi from the Pezizaceae family consistently prevalent across all sites. However, potentially opposing associations to aridity index were observed. Analysis of functional guilds revealed some redundancy in dispersal roles between boodies and woylies when co‐occurring. However, subtle differences in their consumption of secondary fungal resources may promote the dispersal and persistence of a broader range of fungal taxa. Our study reinforces the prevalence of truffle‐like fungi in mycophagous mammal diets and suggests that minor dietary differences may aid in reducing competition while providing some complementary ecosystem benefits. We recommend that future translocations be informed by comprehensive resource and carrying capacity assessments of the translocation site, with releases strategically timed for post‐rainfall periods and monitored across complete drought cycles to ensure successful establishment of boodies and woylies.

## Graphical abstract

This study investigated the fungal dietary preferences of boodies and woylies across Mediterranean, semi‐arid and arid regions of Australia using DNA metabarcoding of scat samples, providing a comprehensive molecular characterisation of mycophagous feeding patterns for these species across broad climate gradients. We identified 379 fungal amplicon sequence variants (ASVs) from scat samples, of which 82 ASVs were identified as macrofungi, likely to be deliberately consumed by boodies and woylies. The results reinforce that mycophagous mammals have a strong preference for truffle‐like fungi and suggest that fine‐scale dietary differences may reduce competition while providing some complementary ecosystem benefits in systems where they co‐exist.

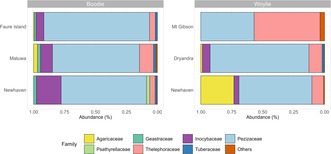

## 1 Introduction

Ecosystem engineers are organisms that play a role in the establishment, modification or maintenance of a habitat through bioturbation (digging activities), structural engineering or trophic impacts (Coggan et al. 2018; Jones et al. 1996). Among these ecosystem engineers, digging mammals represent a guild which is vital for influencing soil properties, plant and fungal communities, as well as providing shelter for other species (Beca et al. 2020; Dundas et al. 2018; Eldridge and Mensinga 2007; Palmer, Beca, et al. 2021; Valentine et al. 2012; Whittington‐Jones et al. 2011). Translocations of digging mammals offer dual benefits. They contribute to the conservation of threatened digging mammal species and the restoration of ecosystems through reinstating ecological processes that have been lost following local extinctions of ecosystem engineers (Palmer et al. 2020). These translocations are especially relevant to Australia's digging mammals, which have experienced the highest rate of decline and extinction globally as a result of predation by introduced cats ( *Felis catus* ) and foxes ( *Vulpes vulpes* ) (Beca et al. 2021; Woinarski et al. 2015).

A subset of Australian digging mammals are mycophagous and are particularly important for the dispersal of hypogeous (truffle‐like) fungi, which have evolved morphologies that rely on their mammalian consumers (Elliott et al. 2022). These hypogeous fungi are predominantly ectomycorrhizal and have mutualistic relationships with plants. In return for a carbon source, the fungi ensure plant nutrient uptake, water movement and provide mycorrhizal feedback networks between plants and fungi (Agerer 2001; Allen 2007; Gorzelak et al. 2015; Peay et al. 2008; Tedersoo et al. 2010; Tedersoo and Smith 2013). Dundas et al. (2018) found that activity from mycophagous digging mammals in a fenced enclosure led to a significantly greater amount of ectomycorrhizal fungi, in contrast to outside the enclosure, where densities of those mammals were low to absent. Loss of these animals can therefore result in detriment to overall ecosystem health (Fleming et al. 2014). Fortunately, previous successes in translocating Australian digging mammals have shown that with careful planning and goal setting, translocations offer the opportunity to restore digging mammal populations and their associated ecosystems (Palmer et al. 2020).

**FIGURE 1:**
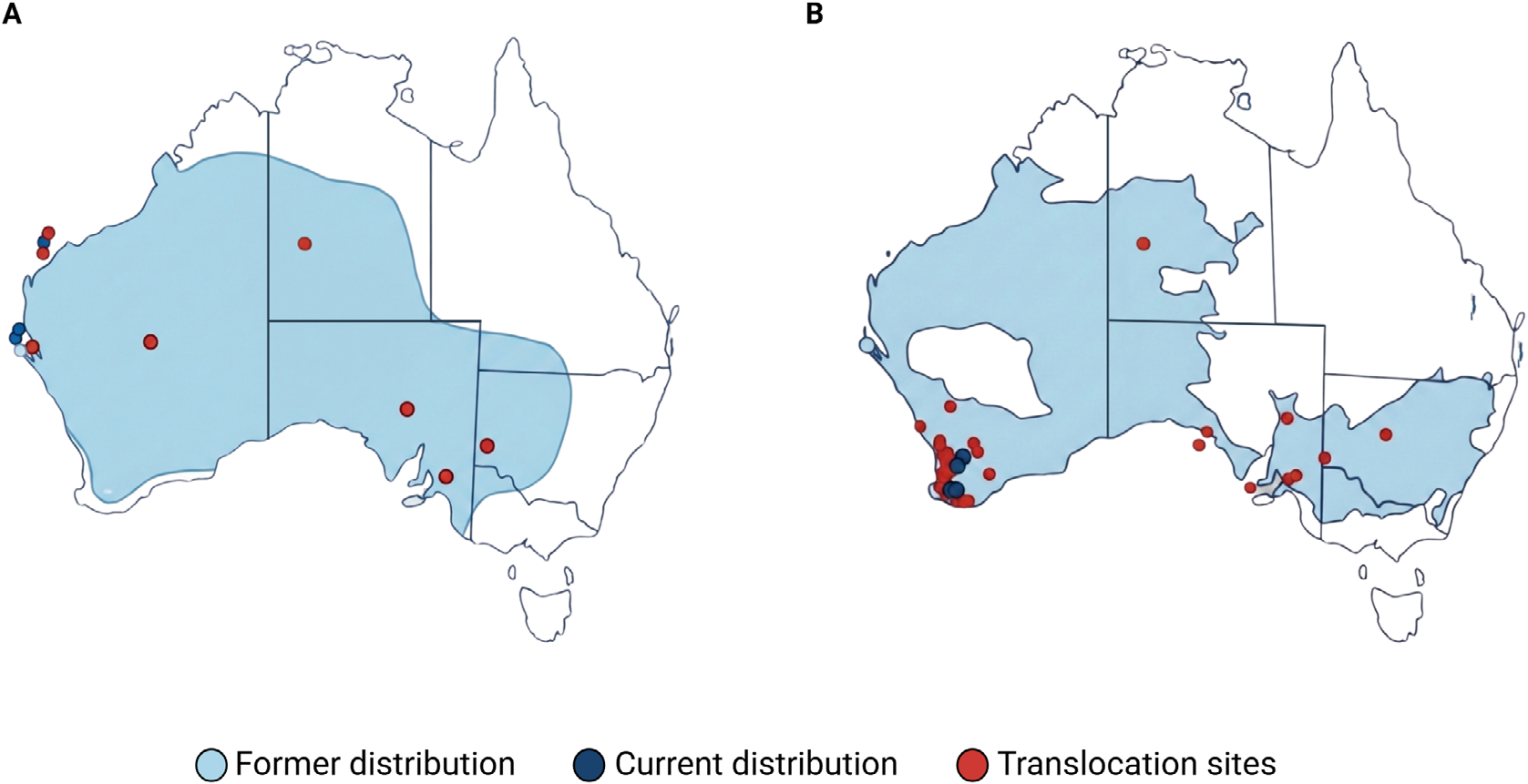
Former, current and translocated distributions of (A) boodies (burrowing bettong; *Bettongia lesueur* ) and (B) woylies (brush‐tailed bettong; *Bettongia penicillata* ) across Australia (adapted from Baker and Gynther (2023)).

The boodie (burrowing bettong; *Bettongia lesueur* ) and woylie (brush‐tailed bettong; *Bettongia ogilbyi*), two medium‐sized Australian marsupials, are ecosystem engineers through their digging behaviours and varying extents of mycophagy (Dundas et al. 2018; Palmer, Valentine, et al. 2021; Robley et al. 2001; Zosky et al. 2018). They differ in their bioturbation activities, with boodies exhibiting semi‐fossorial digging behaviour and woylies functioning primarily as surface foragers (Beca et al. 2021). Both species were historically widespread across mainland Australia and have experienced catastrophic range contractions (Burbidge et al. 1988; Noble et al. 2007; Warburton 2014; Wayne et al. 2013a, 2013b). In contrast to their historically overlapping ranges, contemporary distributions show a clear separation, with remnant and translocated boodie populations occurring primarily in semi‐arid to arid regions, while woylies are predominantly found in temperate, Mediterranean‐type climates across Australia (Figure 1). Co‐existence of both species is now limited to a few translocated sites (Burbidge and Short 2023; De Tores and Marlow 2023). Under the Australian Commonwealth (EPBC Act), boodies and woylies are listed as Vulnerable and Endangered, respectively (Department of Climate Change, Energy, the Environment and Water 2025).

As part of an ecological restoration project managed by the Department of Biodiversity, Conservation and Attractions (DBCA) on Dirk Hartog Island (Wirruwana) in the Shark Bay World Heritage Area of Western Australia, boodies and woylies are planned for reintroduction onto this 63,000 ha semi‐arid island. Dirk Hartog Island once supported populations of both species before their extirpation due to feral cat ( *Felis catus* ) predation and habitat degradation from pastoral activities (Burbidge and George 1978; Morris et al. 2017). Following the successful eradication of cats, sheep and goats, vegetation recovery has created potential habitat for reintroduction (Algar, Johnston, et al. 2020; Algar, Morris, et al. 2020; Heriot et al. 2019). However, due to the cryptic nature of fungi, it is uncertain whether fungal resources have remained through the landscape alterations.

Unlike their more specialised mycophagous relatives in the Potoroidae family, such as Gilbert's potoroo ( *Potorous gilbertii* ) (Nguyen et al. 2005) and long‐footed potoroo ( *Potorous longipes* ) (Bennett and Baxter 1989), boodies and woylies exhibit a higher dietary flexibility in addition to their mycophagous tendencies. Both species have omnivorous feeding strategies that encompass a diverse array of food items, including invertebrates, seeds, bulbs, tubers, other plant material and carrion (Bice and Moseby 2008; Robley et al. 2001; Zosky et al. 2018). Studies have also shown that they can shift their consumption patterns seasonally and spatially in response to resource availability (Bice and Moseby 2008; Zosky et al. 2018). This dietary plasticity has likely contributed to their historical success across diverse Australian landscapes. However, in the context of translocations where animals are under an inevitable level of stress, there is an impetus to ensure that appropriate food resources are available, as there is a narrow window between animal release and potential starvation if resources are not quickly identified and located (Bannister et al. 2021; Dickens et al. 2010). Achieving this requires detailed dietary characterisation for both species.

Here, we employ DNA metabarcoding techniques to study the fungal consumption patterns of these bettongs across a climate gradient encompassing Mediterranean‐type, semi‐arid and arid climate regions. While previous studies (Bice and Moseby 2008; Robley et al. 2001; Zosky et al. 2018) have characterised the broader dietary patterns of boodies and woylies, little is known about their mycophagous diets across different climate types, particularly in semi‐arid and arid environments similar to Dirk Hartog Island. Former studies also relied on microscopic examinations of undigested material in scats (Bice and Moseby 2008; Robley et al. 2001; Zosky et al. 2018), which have significant limitations for fungal dietary analysis due to large proportions of undescribed fungal taxa (Mueller et al. 2007). By choosing to use DNA metabarcoding techniques, we anticipated the identification of a greater number of fungi by reference to fungal sequence libraries (Cloutier et al. 2019; Hopkins et al. 2021; Nuske et al. 2019). We applied this method to boodie and woylie scat samples, resulting in high‐resolution taxonomic identification of consumed fungi and valuable insights into local fungal communities. These mammals serve as effective biological samplers of cryptic fungi, particularly hypogeous (underground) species that are difficult to detect through conventional survey methods which are labour intensive and time consuming. Understanding the dietary overlap between boodies and woylies is also critical, given the scarcity of sites where the two species currently coexist (Burbidge and Short 2023; De Tores and Marlow 2023). Moreover, the failure of some translocation attempts to establish sympatric populations could potentially be due to intensified interspecific competition during resource‐limited periods such as droughts, when dietary niches may converge (Hayward et al. 2010).

Developing a more detailed profile of their fungal consumption patterns would not only illuminate potential dietary limitations but also clarify their role in fungal dispersal and shaping broader ecosystem health across varied climatic conditions. We address these objectives through the following research questions: (1) What fungal taxa do the diets of boodies and woylies include? (2) How do their mycophagous diets differ, and what is the potential for interspecific resource competition? (3) How do mycophagous diets vary across aridity index gradients? (4) What ecological functions can these species provide through their mycophagous activities? These findings will help to guide conservation management decisions for boodies and woylies, as well as provide insight into the potential for ecosystem restoration, particularly for the Dirk Hartog Island reintroductions.

## 2 Methods

### 2.1 Study Sites and Sample Collection

**FIGURE 2:**
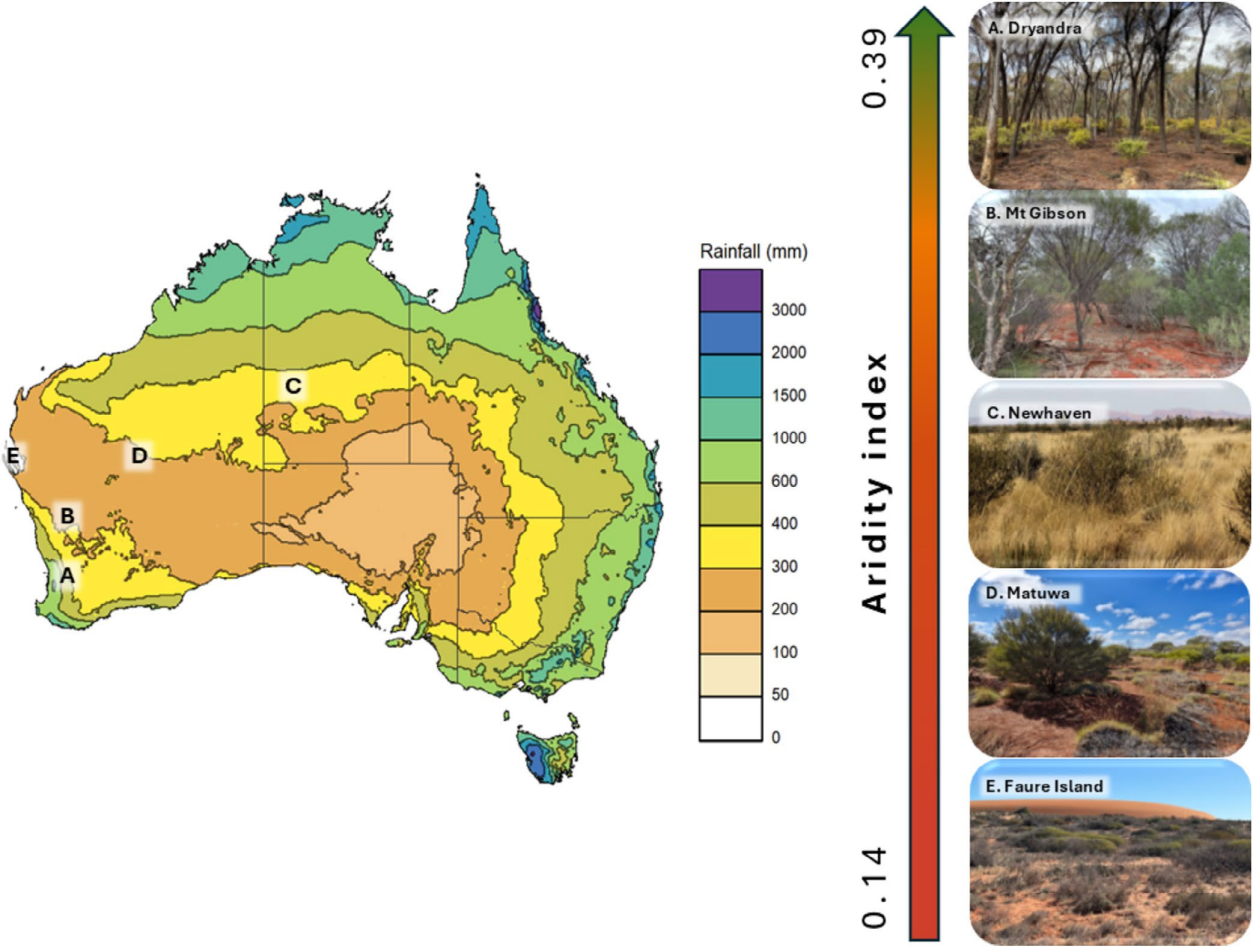
Study sites are numbered on the map of mean annual rainfall and along the aridity gradient from the most mesic (A) to the most arid (E), based on a standard 30‐year climatology (1995–2025) (Bureau of Meteorology, 2025). Aridity index (AI) in Dryandra: 0.39; Mt. Gibson: 0.24; Newhaven: 0.23; Matuwa: 0.17; and Faure Island: 0.14.

Five sites across Western Australia and the Northern Territory were selected to represent a range of climate types (Figure 2). We employed aridity index (AI) as a climate gradient measure across study sites, where 0 represents high aridity and 1 represents low aridity, as it integrates rainfall and moisture parameters that are key determinants of fungal community composition and productivity (Decker et al. 2023; Deslippe 2024; Maestre et al. 2015; Trappe et al. 2008). Faure Island Wildlife Sanctuary (Faure Island) is a 4554 ha introduced predator‐free safe haven managed by Australian Wildlife Conservancy (AWC), representing an arid population (AI = 0.14) of translocated boodies (Australian Wildlife Conservancy 2025a). Matuwa Kurrara Kurrara National Park (Matuwa) represents an arid population (AI = 0.17) of translocated boodies that reside in a 1090 ha introduced predator‐free fenced area in the park (Lohr 2019). Dryandra Woodland National Park (Dryandra) spans 28,066 ha in a Mediterranean‐type climate (AI = 0.39) and represents one of the last natural remnant populations of woylies (Wayne et al. 2013b). Both Matuwa and Dryandra are managed by DBCA. Mt. Gibson Wildlife Sanctuary (Mt Gibson) represents a semi‐arid population (AI = 0.24) of translocated woylies that reside in a 7838 ha introduced predator‐free fenced area in the sanctuary (Australian Wildlife Conservancy 2025b). Lastly, Newhaven Wildlife Sanctuary (Newhaven) represents semi‐arid (AI = 0.23) translocated populations of boodies and woylies which reside in a 9400 ha introduced predator‐free fenced area in the sanctuary (Australian Wildlife Conservancy 2025c). Both Mt. Gibson and Newhaven are managed by AWC.

**FIGURE 3:**
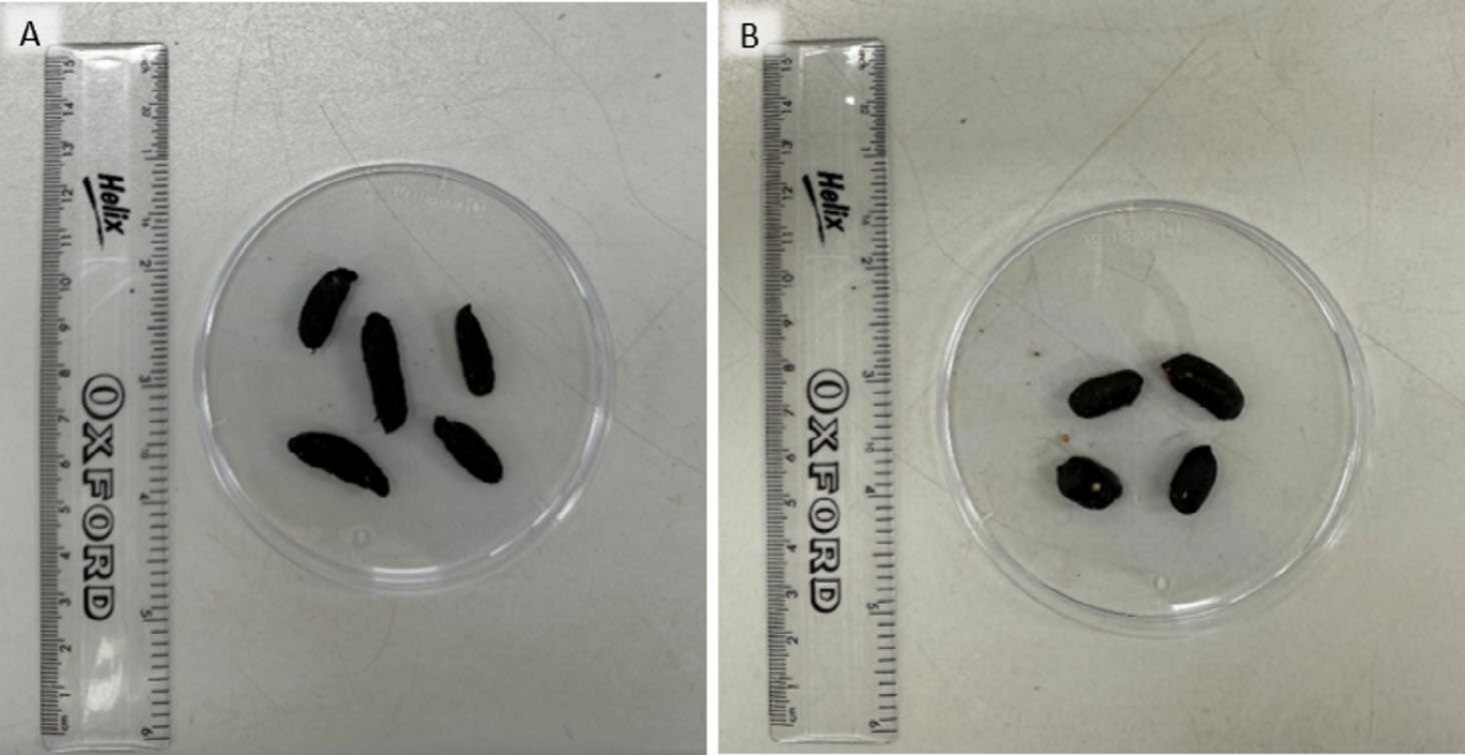
(A) Boodie and (B) woylie scats collected from traps for dietary analysis.

Boodie and woylie scats were collected during ongoing monitoring programs by AWC and DBCA, which included trapping animals. Boodie scats were collected from Faure Island, Matuwa and Newhaven in the autumn of 2024. Woylie scats were collected from Mt. Gibson and Newhaven in the autumn of 2024 and Dryandra in the autumn of 2018. An autumn sample collection was chosen as fungal abundance and consumption by mycophagous mammals are thought to be highest during these months (Christensen 1980; Nguyen et al. 2005; Połatyńska‐Rudnicka 2014; Zosky et al. 2018). Scats were collected fresh from traps that animals had been caught in, ensuring the correct identification of species' scats (Figure 3). Fifteen samples, consisting of approximately five faecal pellets per sample, were collected per location and species and stored in sterile vials. Due to the remote nature of all study sites and logistical constraints, samples were stored chilled until they arrived in Perth, where they were moved into a −20°C freezer until ready to be processed in the laboratory.

### 2.2 DNA Extraction and Sequencing

Scat samples were individually homogenised, and 0.25 g subsamples were taken for DNA extraction using the DNeasy PowerLyzer PowerSoil DNA extraction kit (Qiagen) as per the manufacturer's instructions. Quality control measures, including extraction and PCR negative controls, were included to ensure no contamination occurred during DNA extraction and amplification. Extracted DNA concentration was measured using a NanodropOne spectrophotometer (ThermoFisher), ensuring minimum thresholds (5 ng/μL) were met in all samples, and no detectable DNA was present in controls. We employed the single‐marker ITS2 metabarcoding region for PCR following the methodology described in Brace et al. (2024), which used two primers: a fungal‐specific primer fITS7 (TACGGTAGCAGAGACTTGGTCTGGGTGARTCATCGAATCTTTG; Ihrmark et al. 2012) and a general primer ITS4 with adapters (ACACTGACGACATGGTTCTACACGCCTSCSCTTANTDATATGC; White et al. 1990), performed using AmpliTaq Gold (ThermoFisher) in replicates. While using a multi‐marker approach might substantiate taxonomic resolution, the approach also scales laboratory efforts, sequencing, analysis and costs significantly (Tedersoo et al. 2022; Watts et al. 2026). Furthermore, the use of ITS2 is standard practice with comparable studies (Dundas et al. 2018; McIntyre et al. 2026; Tay et al. 2018), as the marker yields a higher number of taxonomic units and phylogenetic richness for fungi than ITS1 and focuses on higher fungi such as those likely to form macroscopic fruitbodies (Tedersoo and Lindahl 2016). A sample library was compiled from similar concentrations of each sample and sequenced using Illumina MiSeq technology at the eDNA Frontiers laboratory in Curtin University.

### 2.3 Bioinformatics

Raw fungal ITS sequences were demultiplexed using the ‘insect’ package (Wilkinson et al. 2018) and processed through the DADA2 bioinformatics pipeline (Callahan et al. 2016) in R 4.1.0 programming language (R Core Team 2020) and RStudio 4.4.1 (R Core Team 2024). Sequence reads were trimmed, filtered and inspected for quality based on read quality profiles. Fungal DNA metabarcoding inherently produces highly variable read depth distributions across samples, and while rarefaction or coverage‐based normalisation may address sequencing depth variation, we found that rarefaction of our dataset to the minimum sample depth would have resulted in the unnecessary elimination of genuine sequences and substantial loss of information (McMurdie and Holmes 2014). Instead, the DADA2 algorithm we employed uses sample‐specific error models to distinguish true biological variants from sequencing errors, providing robust sequence variant identification that is less susceptible to technical artefacts than traditional taxonomic unit clustering approaches (Callahan et al. 2016). Sequences with ITS merged pairs shorter than 50 base pairs were excluded. Sequence primers were removed using the Cutadapt tool (Martin 2011), and remaining sequences were clustered at the nucleotide level to identify amplicon sequence variants (ASVs). As per the DADA2 methodology, any bimeric, chimeric, and singleton ASVs were removed using nochim and denovo tools (Callahan et al. 2016). Putative taxonomy was assigned to the ASVs based on the closest match in the custom‐curated UNITE database (UNITE general FASTA release for Fungi 2 v10.0; Abarenkov et al. 2024). Sequence reads were matched to their respective references if they could be identified at the very least to the Kingdom level. Reads that failed to be identified at the Kingdom level were removed from downstream analysis.

As per FUNGuild (Nguyen et al. 2016), fungal ASVs were categorised by putative life history using ecological functional guilds where possible. For this study, we included the following guilds as per Hopkins et al. (2024): arbuscular mycorrhizal, ectomycorrhizal, endophyte, ericoid mycorrhizal, other symbiotrophs, animal pathogens, fungal parasites, lichen parasites, plant pathogens, other pathotrophs, litter saprotrophs, wood saprotrophs, other saprotrophs, and unknown function (ASVs without a clear function). ASVs with multiple guild assignments contributed to the counts of all relevant functional guilds. Raw sequence data is stored in the Sequence Read Archive (SRA) curated by NCBI under the accession number PRJNA1360078. The ‘phyloseq’ package (McMurdie and Holmes 2013) was then used to integrate a sample matrix with the sequence table and taxonomic assignment output from DADA2 to facilitate species, site and sample‐level comparisons. Only ASVs with more than five reads in at least 5% of the samples were retained using the ‘prune_taxa’ tool in Phyloseq. This threshold further differentiates rare, potentially spurious sequences that are most likely to represent technical artefacts or contamination while retaining biologically meaningful variants (Brace et al. 2024; Hopkins et al. 2024).

### 2.4 Statistical Analyses

From the output of our bioinformatics analysis, 379 fungal ASVs were derived. We excluded microfungi, yeasts, and pathogens from subsequent dietary comparisons between species using the ‘subset_taxa’ function in Phyloseq, as these taxa likely represent incidental consumption, environmental contamination, or components of the gut microbiota rather than deliberate dietary choices, further reducing noise in the dataset. To assess fungal diets of boodies and woylies across the five study sites, we calculated Shannon's diversity index and observed species richness of ASVs using the ‘estimate_richness’ function in Phyloseq. Generalised linear mixed effect models were then applied to analyse these Alpha diversity metrics using the ‘glmmtmb’ package (Brooks et al. 2017) and Gaussian distribution. We visually inspected residuals with the ‘dharma’ package (Hartig and Hartig 2020). For all analyses, we utilised a *p* value of < 0.05 to indicate statistical significance and data visualisation was performed using the package ‘ggplot2’ (Wickham and Chang 2014).

We examined dietary composition variations between boodies and woylies across study sites using nonmetric multidimensional scaling (NMDS) analysis based on Bray–Curtis dissimilarity, calculated from ASV species matrix relative abundance data. This inherently controls for differences in total read depth across samples by converting counts to proportions. To complement the NMDS and quantitatively assess community composition differences, we performed a permutational multivariate analysis of variance (PERMANOVA) (Anderson 2001) using the ‘adonis’ function from the ‘vegan’ package (Dixon 2003). Pairwise PERMANOVA tested a sample matrix against species and site, stratified using 999 permutations. To supplement our understanding of the ecological roles that boodies and woylies play, we calculated the relative abundance of fungal functional guilds consumed by each species.

## 3 Results

### 3.1 Fungal Taxa Present in Scats

Collectively, the boodie and woylie scat samples yielded 379 fungal ASVs. These consisted of the fungal phyla Ascomycota (72.4%), Basidiomycota (16.0%), Glomeromycota (10.4%), Chytridiomycota (0.4%), Mortierellomycota (0.2%), Calcarisporiellomycota (0.1%) and unassigned phyla (0.4%). After excluding fungi deemed accidental consumption or environmental contaminants, 82 ASVs (75.5% Ascomycota; 24.5% Basidiomycota) remained. The 45 boodie scat samples yielded 67 fungal ASVs, with each scat containing an average of 5.13 ± 2.58 (mean ± SD) ASVs per sample (range 2–10). The 45 woylie scat samples yielded 51 fungal ASVs, with each scat containing an average of 5.87 ± 2.62 (mean ± SD) ASVs per sample (range 2–13). It was possible to assign 28.6% to known species, 97.5% to genus, and 100% to family (Table 1). We used the assigned fungal ASVs from scat samples to infer the diet of the mammals (hereafter referred to as “diet”), with the caveat that more digestible fungal species could be underrepresented in the scat samples.

**TABLE 1:** Families and genera of fungi identified from boodie and woylie scat samples.

Boodie		Woylie	
Family	Genus	Family	Genus
Agaricaceae	*Tulostoma*	Agaricaceae	*Tulostoma*
	*Lepiota*		*Lepiota*
	Unidentified 1	Entolomataceae	*Clitopilus*
	Unidentified 2	Geastraceae	*Geastrum*
Bolbitiaceae	*Conocybe*	Inocybaceae	*Inocybe*
Entolomataceae	*Clitopilus*		*Mallocybe*
Geastraceae	*Geastrum*	Pezizaceae	*Phaeopezia*
Inocybaceae	*Inocybe*		*Ruhlandiella*
	*Mallocybe*	Pyronemataceae	*Moscella*
Pezizaceae	*Phaeopezia*	Thelephoraceae	*Thelephora*
	*Ruhlandiella*		*Tomentella*
Psathyrellaceae	*Coprinellus*	Tuberaceae	*Dingleya*
Pyronemataceae	*Moscella*		*Reddellomyces*
Thelephoraceae	*Thelephora*	Xylariaceae	*Xylaria*
	*Tomentella*		
Tuberaceae	*Dingleya*		
	*Reddellomyces*		

**FIGURE 4:**
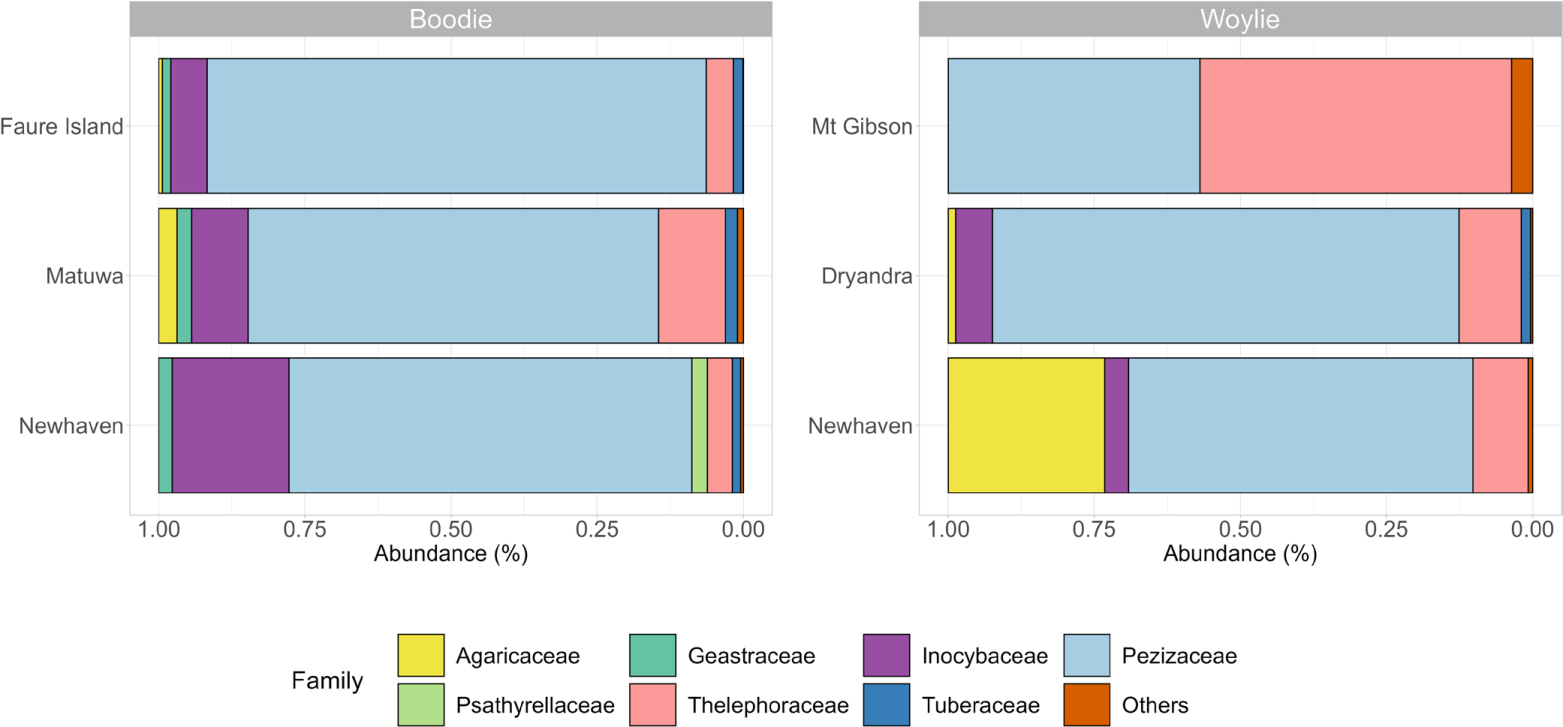
Average abundance of fungal family in the diet of boodies on Faure Island, Matuwa and Newhaven; and woylies in Mt. Gibson, Dryandra and Newhaven; after excluding fungi deemed accidental consumption and environmental contaminants.

Fungi from the family Pezizaceae formed a large percentage of the diet of boodies and woylies at all study sites (85.4% of boodie diet on Faure Island, 70.3% of boodie diet at Matuwa and 68.9% of boodie diet at Newhaven; 79.8% of woylie diet at Dryandra, 43.1% of woylie diet at Mt. Gibson and 59.0% of woylie diet at Newhaven) (Figure 4). These were predominantly comprised of ASVs putatively assigned to the genus *Ruhlandiella*. Thelephoraceae species were also observed in the diets of boodies and woylies across all study sites, with the highest relative abundance observed in woylie diets at Mt. Gibson (53.3%). Inocybaceae was another common fungal family observed in the diet of boodies at all study sites and woylies in Dryandra and Newhaven, albeit comprising a smaller relative abundance of their diet (Figure 4). At Newhaven, the sole study site supporting both species, boodies demonstrated higher relative consumption of Inocybaceae (20.0%) compared to woylies (4.08%). Conversely, consumption of Agaricaceae fungi at Newhaven was exclusive to woylies (26.8%) and entirely absent from boodie scat samples despite being present in small relative abundance in the diet of boodies on Faure Island (0.640%) and at Matuwa (3.10%) (Figure 4).

### 3.2 Richness and Shannon's Diversity

**FIGURE 5:**
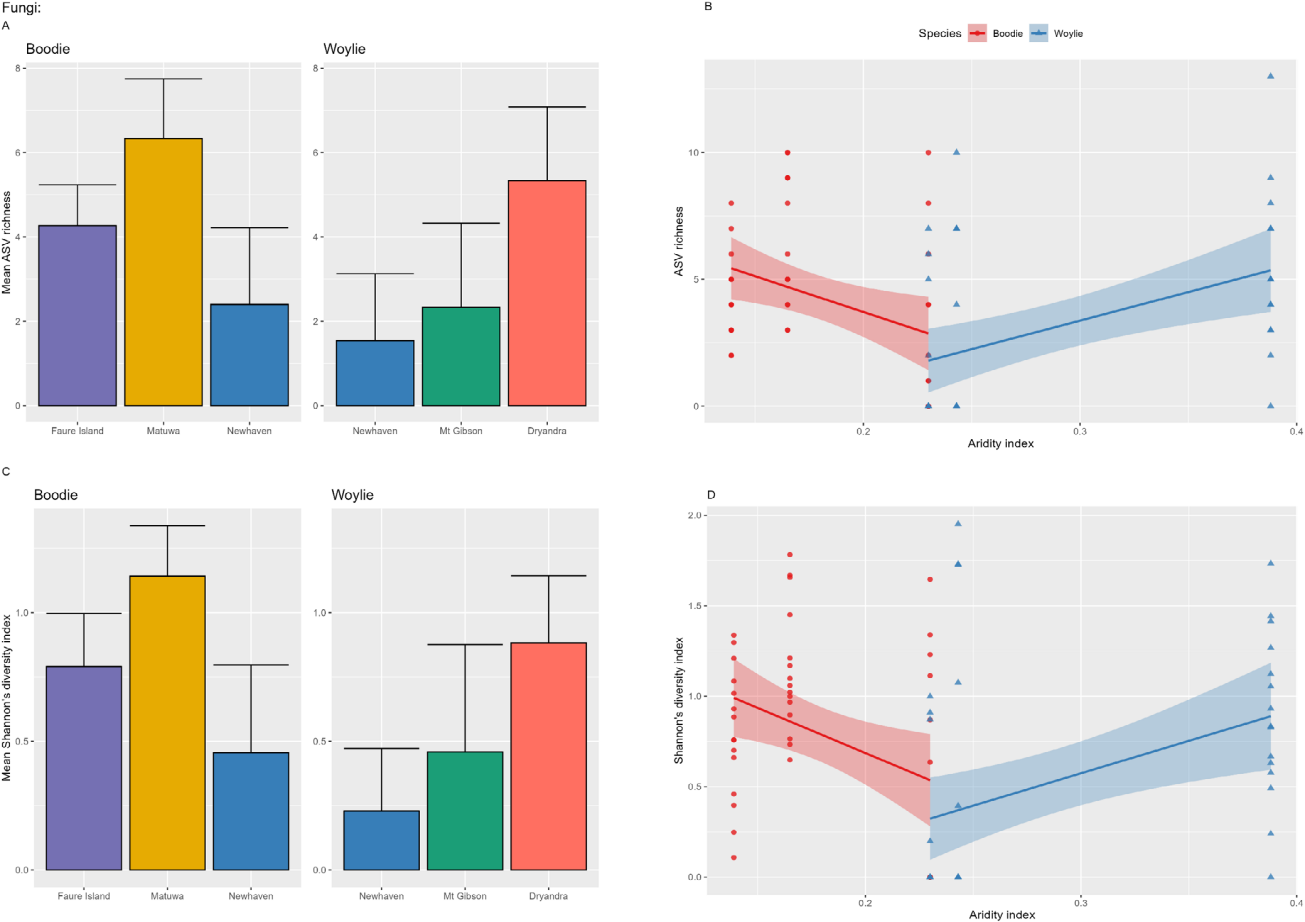
Alpha diversity metrics of fungi in the diet of boodies on Faure Island, Matuwa and Newhaven; and woylies in Mt. Gibson, Dryandra and Newhaven. (A) Mean fungal ASV richness with 95% confidence intervals and (B) raw values of ASV richness with fitted linear regression lines and confidence interval bands. (C) Mean Shannon's diversity with 95% confidence intervals and (D) raw values of Shannon's diversity with fitted linear regression lines and confidence interval bands. Aridity index (AI) in Dryandra: 0.39; Mt. Gibson: 0.24; Newhaven: 0.23; Matuwa: 0.17; and Faure Island: 0.14.

Overall fungal ASV richness between the diet of boodies (estimate = 4.33, SE = 0.49, *z* = 8.88) and woylies (estimate = 3.14, SE = 0.70, *z* = 7.17, *p* = 0.091) show no significant differences. However, overall fungal diversity in the diet of boodies (estimate = 0.80, SE = 0.09, *z* = 9.25) appear to be significantly higher than woylies (estimate = 0.54, SE = 0.12, *z* = 7.15, *p* = 0.038). Fungal ASV richness and diversity in boodie diets varied significantly among study sites (*F*_2,42_ = 8.53, *p* < 0.001 and *F*_2,42_ = 8.25, *p* < 0.001), with Newhaven supporting the lowest values (estimate = 2.40, SE = 0.67, *z* = 3.56, *p* = 0.001 and estimate = 0.46, SE = 0.12, *z* = 3.81, *p* < 0.001) compared to Matuwa (estimate = 6.33, SE = 0.95, *z* = 4.13, *p* < 0.001 and estimate = 1.15, SE = 0.17, *z* = 4.06, *p* < 0.001), while Faure Island showed nonsignificant increases for both metrics (estimate = 4.27, SE = 0.95, *z* = 1.96, *p* = 0.057 and estimate = 0.79, SE = 0.17, *z* = 1.98, *p* = 0.054) (Figure 5). Similarly, woylie fungal dietary richness and diversity differed significantly across sites (*F*_2,40_ = 5.71, *p* = 0.007 and *F*_2,40_ = 4.79, *p* = 0.014), with Newhaven supporting the lowest values (estimate = 1.54, SE = 0.88, *z* = 1.75, *p* = 0.089 and estimate = 0.23, SE = 0.16, *z* = 1.45, *p* = 0.155) compared to Dryandra which exhibited significantly higher values (estimate = 5.33, SE = 1.20, *z* = 3.15, *p* = 0.003 and estimate = 0.88, SE = 0.22, *z* = 3.02, *p* = 0.004), whereas Mt. Gibson showed no significant difference to Newhaven for either metric (estimate = 2.33, SE = 1.20, *z* = 0.66, *p* = 0.513 and estimate = 0.46, SE = 0.22, *z* = 1.06, *p* = 0.294). Within Newhaven, no significant differences were observed between the richness and diversity of fungal diets between boodies (estimate = 2.40, SE = 0.78, *z* = 3.09 and estimate = 0.46, SE = 0.14, *z* = 3.34) and woylies (estimate = 1.54, SE = 1.14, *z* = 2.34, *p* = 0.456 and estimate = 0.23, SE = 0.20, *z* = 2.21, *p* = 0.268) (Figure 5a,c). The Alpha diversity indices showed potentially opposing associations to the aridity index between the two species. A negative relationship was observed for boodies (red), while a positive relationship was observed for woylies (blue) (Figure 5b,d). However, site‐specific and temporal factors cannot be fully disentangled from aridity effects with our current study design.

### 3.3 Community Composition

**FIGURE 6:**
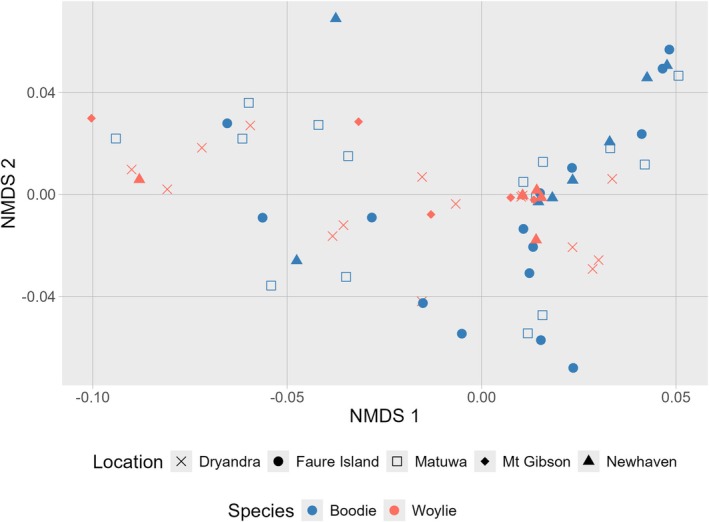
Bray Curtis dissimilarity NMDS for boodies on Faure Island, Matuwa and Newhaven; and woylies in Mt. Gibson, Dryandra and Newhaven.

The NMDS ordination revealed distinct fungal dietary community compositions between boodies and woylies (stress = 0.159) (Figure 6), with PERMANOVA confirming significant overall differences in mycophagous diet composition between the two species (*F*_1,86_ = 4.44, *R* ^2^ = 0.049, *p* = 0.001). Pairwise comparisons within each species demonstrated significant geographic variation in fungal consumption patterns, with boodie diets differing significantly between Matuwa and Newhaven (*F* = 3.00, *R* ^2^ = 0.097, *p* = 0.001) and between Faure Island and Newhaven (*F* = 2.77, *R* ^2^ = 0.090, *p* = 0.008), but not between Faure Island and Matuwa (*F* = 1.03, *R* ^2^ = 0.036, *p* = 0.391). Similarly, woylie fungal dietary compositions varied significantly across study sites, with both Dryandra versus Mt. Gibson (*F* = 5.02, *R* ^2^ = 0.15, *p* = 0.002) and Dryandra versus Newhaven (*F* = 4.95, *R* ^2^ = 0.16, *p* = 0.001) showing distinct community structures, while Mt. Gibson and Newhaven exhibited similar fungal consumption patterns (*F* = 0.51, *R* ^2^ = 0.019, *p* = 0.848). Within Newhaven, boodie and woylie were not significantly different (*F* = 1.29, *R* ^2^ = 0.047, *p* = 0.137).

### 3.4 Functional Groups

**FIGURE 7:**
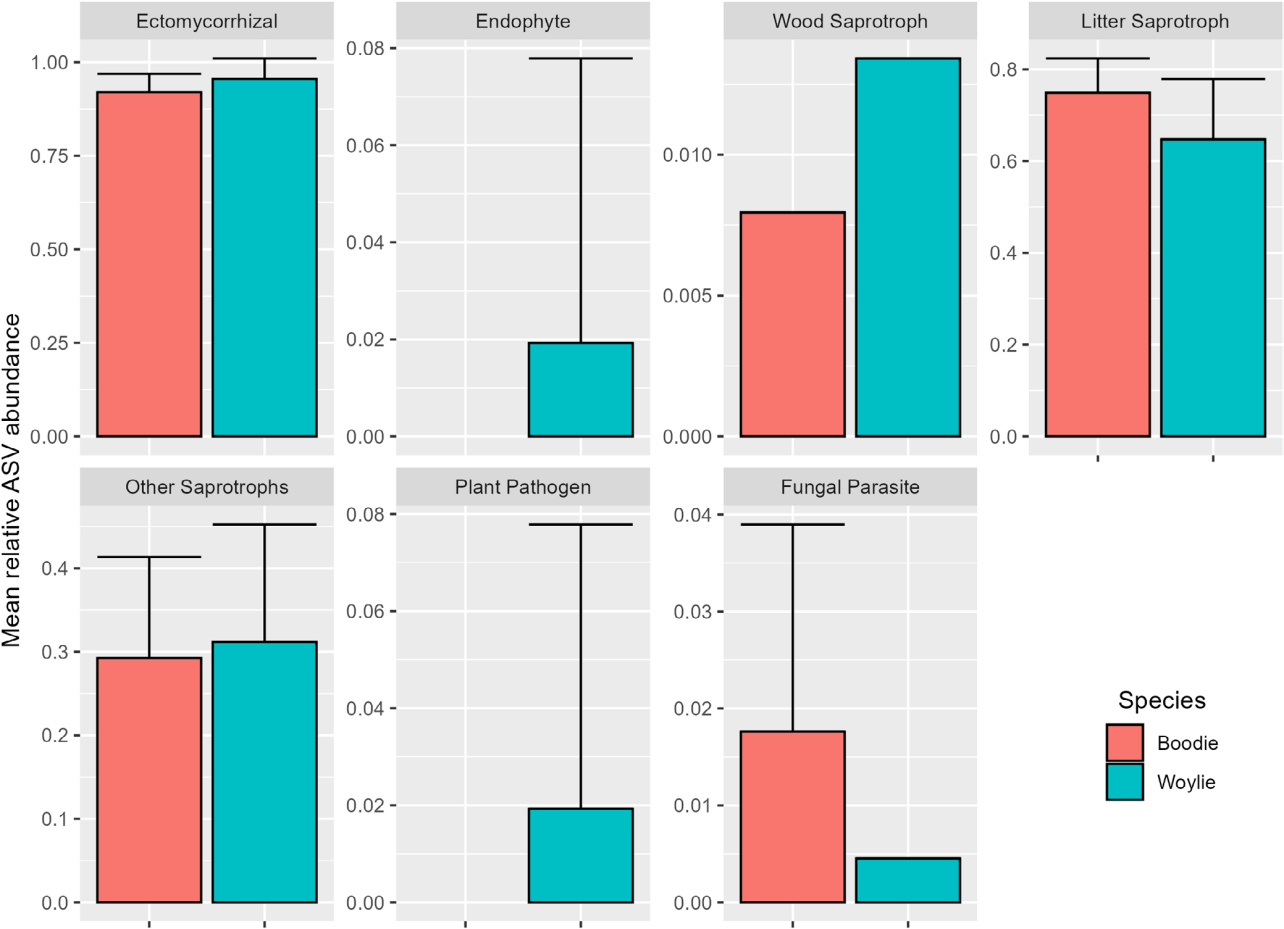
Fungal functional guilds for the diet of boodies on Faure Island, Matuwa and Newhaven; and woylies in Mt. Gibson, Dryandra and Newhaven. Values are means with 95% confidence intervals.

**FIGURE 8:**
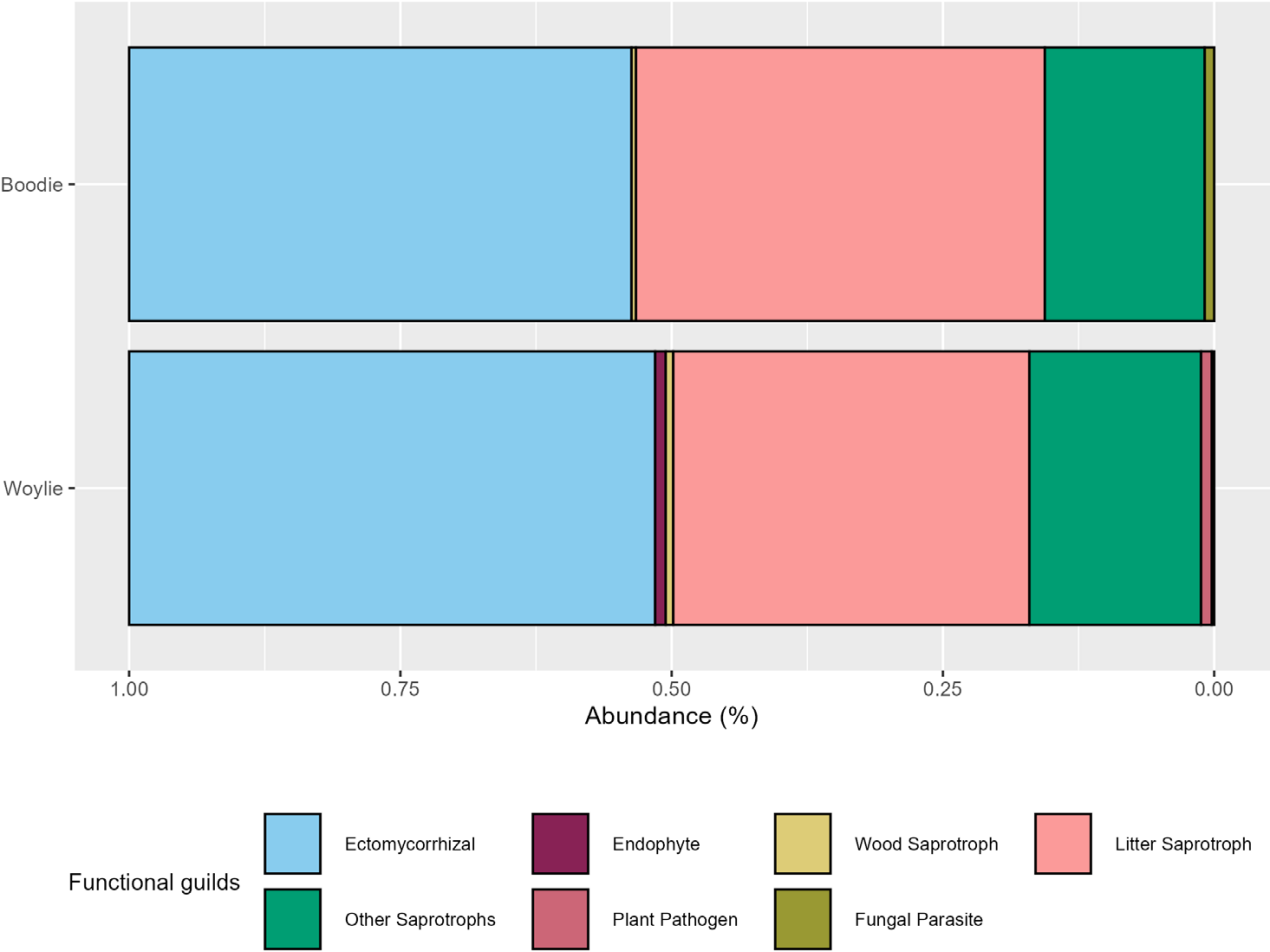
Average abundance of fungal functional guilds in the diet of boodies on Faure Island, Matuwa and Newhaven; and woylies in Mt. Gibson, Dryandra and Newhaven.

Analysis of functional guild composition revealed that both boodies and woylies consumed predominantly ectomycorrhizal fungi (e.g., Inocybaceae, Pezizaceae, Thelephoraceae and Tuberaceae), with no significant difference between species (boodies: estimate = 0.92, SE = 0.023, *z* = 40.2, *p* < 0.001; woylies: estimate = 0.97, SE = 0.037, *z* = 0.96, *p* = 0.340) (Figures 7, 8). Litter saprotrophic fungi (e.g., Pezizaceae) comprised the second most abundant guild in both species' diets, though boodies showed slightly higher consumption rates than woylies (boodies: estimate = 0.75, SE = 0.045, *z* = 16.6, *p* < 0.001; woylies: estimate = 0.65, SE = 0.069, *z* = −1.47, *p* = 0.147) (Figures 7, 8). Other saprotrophic fungi (e.g., Agaricaceae, Geastraceae and Psathyrellaceae) and fungal parasites (e.g., Entolomataceae) were consumed in relatively minor proportions by both species, with no significant interspecific differences detected (other saprotrophs—boodies: estimate = 0.29, SE = 0.058, *z* = 5.07, *p* < 0.001; woylies: estimate = 0.019, SE = 0.31, *z* = 0.21, *p* = 0.831; fungal parasites—boodies: estimate = 0.018, SE = 0.0077, *z* = 2.29, *p* = 0.084; woylies: estimate = 0.005, SE = 0.019, *z* = −0.69, *p* = 0.526), while wood saprotrophic fungi were rarely encountered in either species' diet. Endophytes (e.g., Xylariaceae) and plant pathogens (e.g., Xylariaceae) were detected exclusively in woylie diets, though both guilds occurred in similarly low proportions (Figures 7, 8).

## 4 Discussion

Mycophagous digging mammals such as boodies and woylies are important ecosystem engineers that have been lost from much of their former range across Australia (Burbidge et al. 1988; Palmer, Beca, et al. 2021; Warburton 2014; Zosky et al. 2018), prompting reintroduction efforts that aim to achieve both species conservation and broader ecosystem restoration goals. In this study, we used DNA metabarcoding approaches to investigate the mycophagous diets of these species, providing critical baseline information to support their ongoing reintroductions. Specifically, we examined fungal dietary composition, interspecific dietary differences and competition potential, observed responses to aridity index gradients, and the ecosystem restoration functions these mycophagous mammals may provide.

### 4.1 Mycophagous Diets of Boodies and Woylies

Boodies are opportunistic mycophagists (Claridge and Trappe 2005; Nuske et al. 2017), with previous research demonstrating highly variable fungal consumption ranging from 0.02% of their diet at Arid Recovery in northern South Australia (Bice and Moseby 2008) up to 23% at Heirisson Prong in Shark Bay, Western Australia (Robley et al. 2001). On the other hand, woylies function as preferential mycophagists (Claridge and Trappe 2005; Nuske et al. 2017), with fungi comprising between 54% of their diet at Karakamia Sanctuary in south‐west Western Australia to 76% of their diet in Dryandra (Zosky et al. 2018), and a consistently high dependence on fungi year‐round (Christensen 1980). As a result of this fundamental difference in dietary dependence on fungi, the mycophagous diet of woylies has been more extensively studied than that of boodies. Previous research on the diet of woylies in five subpopulations in the south‐west of Western Australia documented remarkable fungal diversity, identifying 79 different fungal spore classes representing at least 15 genera from 14 families through fine fraction analysis (Zosky et al. 2018), while there has been little to no attempt to identify fungal taxa consumed by boodies (Bice and Moseby 2008; Robley et al. 2001; Treloar 2022). Consequently, substantial knowledge gaps remain regarding the comparative mycophagous ecology of these species and their potential for resource competition when co‐occurring.

Our analysis of the bettong scats revealed that fungi from the family Pezizaceae play a significant role in the diets of both boodies and woylies across all study sites. Pezizaceae and Tuberaceae, also present in the bettong diets, include a range of desert truffles (Trappe et al. 2008), reinforcing the importance of hypogeous fungi in the diets of these mycophagous mammals (Claridge and May 1994; Elliott et al. 2022). Fungi from the families Thelephoraceae and Inocybaceae are important secondary fungal resources for boodies and woylies. Thelephoraceae are characterised by their resupinate and thin basidiomata (Zhang et al. 2024), and Inocybaceae are characterised as gilled mushroom‐forming fungi (Matheny and Bougher 2017). Contrary to expectations, our findings revealed that overall, Shannon's diversity was significantly greater in the fungal diets of boodies than woylies, while differences in richness were non‐significant between species. This finding is surprising, as preferential mycophagists would typically be expected to consume a greater diversity of fungi compared to opportunistic mycophagists (Nuske et al. 2018; Quah et al. 2025). However, it is not totally unprecedented, as Nuske et al. (2017) demonstrated that in certain communities, opportunistic mycophagists, such as bush rats ( *Rattus fuscipes* ), can exhibit fungal dietary diversity comparable to that of preferential mycophagists, like the long‐nosed potoroo ( *Potorous tridactylus* ). Further, it is important to consider that this pattern may be reflecting confounding site‐specific effects rather than true interspecific differences, as the species were sampled at largely different locations, with boodies exclusive to Faure Island and Matuwa, and woylies exclusive to Mt. Gibson and Dryandra.

### 4.2 Dietary Overlap and Potential for Competitive Interactions

In Newhaven, where both boodies and woylies have been reintroduced, diversity indices and multivariate analysis revealed no significant overall differences in fungal dietary composition between the two species. This is supported by the findings of Pizzuto et al. (2007), who found that the two species foraged in similar microhabitats, suggesting dietary overlap. Additionally, the consistently low fungal dietary richness and diversity observed in both bettong species at Newhaven suggests that environmental conditions at this site may have constrained foraging opportunities, potentially limiting both species to a reduced subset of fungal resources. Importantly, while Newhaven has a higher aridity index score than Matuwa and Faure, this index considers only the ratio of long‐term average precipitation to potential evapotranspiration, excluding critical factors such as the periodicity of rainfall events that may influence fungal fruiting and availability (Trappe et al. 2008). Despite both species' diets being dominated by Pezizaceae fungi, we observed small differences in the consumption of secondary fungal families. Most notably, Agaricaceae consumption was exclusive to woylies at Newhaven despite being present in boodie diets at other sites where woylies were absent. Conversely, boodies demonstrated consistently higher consumption of Inocybaceae and maintained exclusive access to Geastraceae, Psathyrellaceae and Tuberaceae at this site, potentially reflecting the fungal preferences of boodies and woylies. These patterns demonstrate subtle but ecologically meaningful partitioning between the sympatric populations, indicating that despite broadly similar dietary requirements, the species can coexist through exploiting various secondary fungal resources, which may facilitate reducing competition for identical food sources.

Conservation translocations frequently utilise closed environments like islands and fenced sanctuaries to protect threatened species (Legge et al. 2018), but these restricted systems inherently limit resource availability and prevent natural population regulation mechanisms such as dispersal to alternative foraging areas. Previous research has demonstrated that restricted environments can lead to resource overexploitation, population crashes, and negative impacts on sympatric fauna through interspecific competition (Hayward et al. 2010; Moseby et al. 2018; Treloar et al. 2021). The fundamental difference between opportunistic and preferential mycophagous strategies has important implications for how these species respond to seasonal resource fluctuations and potential competitive interactions. As opportunistic mycophagists, boodies can substantially shift their dietary reliance when fungal abundance declines. Robley et al. (2001) documented reduced fungal dependence during summer months when boodies increased consumption of browse, seeds, and carrion. While woylies also exhibit seasonal dietary flexibility and can incorporate alternative food sources, their preferential mycophagous strategy means they maintain consistently high fungal consumption year‐round (Christensen 1980; Zosky et al. 2018), making them more vulnerable to fungal resource limitations during drier seasons. This greater dependence on fungal resources may become problematic when availability is reduced, as woylies may have a more limited capacity to substitute alternative food sources compared to boodies, potentially amplifying even minor competitive pressures from sympatric populations during periods of resource scarcity. Similar competitive concerns involving boodies and sympatric species have been documented at multiple Australian reserves, including Arid Recovery (Bice and Moseby 2008; Moseby et al. 2018), Matuwa (Treloar et al. 2021), and Scotia (Hayward et al. 2010), emphasising the need for careful resource assessment prior to establishing sympatric populations of mycophagous mammals on Dirk Hartog Island.

### 4.3 Fungal Dietary Variations Between Sites and Aridity Index Gradients

Across all study sites, multivariate analysis revealed significant differences in fungal dietary composition between boodies and woylies. However, these patterns appear to be primarily driven by climatic variation rather than inherent species‐specific preferences. Site clustering based on the similarity of their aridity index values was detected, with the lower aridity index locations, Faure Island and Matuwa, exhibiting similar fungal dietary compositions that differed markedly from Newhaven for boodies. Similarly, among woylie sites, lower aridity index locations, Mt. Gibson and Newhaven, showed convergent fungal dietary patterns that contrasted with the mesic Dryandra site.

In terms of diversity, our analysis revealed potentially opposing associations to aridity index between the two species. Unsurprisingly, woylies demonstrated increased fungal dietary diversity as aridity index values increased. However, contrary to expectations, boodies demonstrated increased fungal dietary diversity as aridity index values decreased. Previous research has shown that bettongs generally decrease fungal consumption and shift toward greater reliance on plant material under resource‐limited conditions (Bice and Moseby 2008; Robley et al. 2001; Zosky et al. 2018). The divergent associations may reflect different behavioural adaptations between the opportunistic and preferential mycophagists; however, our ability to interpret these results is limited by our current study design and gaps in fungal sequence databases. Future work should include sampling across multiple years and seasons to provide a more robust understanding of spatial and climatic trends in mycophagous diets. Additionally, support towards fungal taxonomy and the development of more extensive molecular sequence libraries for Australian fungi is essential to providing better taxonomic resolution to support ecological research. The high proportion of fungi that remains undescribed in Australia is a major contributing factor to the fungal distribution knowledge gap. This limits our ability to distinguish between dietary preference (active selection for certain fungi) and dietary availability (consumption simply reflecting what is present in the environment).

Surprisingly, despite differences in aridity index values between sites, bettongs were able to locate a comparable level of fungal diversity at either end of the spectrum, from Faure Island and Matuwa with low aridity index values, to the forested habitat of Dryandra with the highest aridity index values, reinforcing their adaptable nature. This is encouraging, as Faure Island and Dirk Hartog Island share similar aridity index values due to their geographic proximity, suggesting that Dirk Hartog Island has the potential to support a comparable diversity of fungal resources. However, a critical limitation of this study is that fungal abundance and biomass were not quantified at each location. These metrics are essential for estimating the carrying capacity of a habitat and informing the population management of mycophagous species.

Hypogeous fungi in Australia are predominantly ectomycorrhizal, forming symbiotic associations with a range of perennial host plants, including genera such as *Eucalyptus*, *Leptospermum*, *Allocasuarina*, and *Acacia* (Bougher and Lebel 2001; Warcup 1980). While it is generally assumed that Australian desert truffles, like their forest counterparts, similarly associate with perennial ectomycorrhizal plants, evidence from arid environments elsewhere suggests a broader host range. For instance, desert truffles in Kuwait form specialised mycorrhizal relationships with annuals in the Cistaceae family, and the Kalahari truffle can associate with non‐ectomycorrhizal herbaceous species (Trappe et al. 2008). In Australia, desert truffles have been documented across diverse arid habitats, including sandhills, sandplains, limestone‐based dune systems, woodland, shrubland and at the base of rocky hills, typically fruiting in years of sufficient and well‐timed rainfall. Their phenology is strongly linked to episodic soil moisture, with fruiting bodies often developing rapidly following rain events (Trappe et al. 2008).

### 4.4 Ecological Functions of Bettongs

Boodies and woylies are likely to function as essential fungal vectors, with their high consumption of ectomycorrhizal fungi reiterating the strong association between this fungal guild and mammalian mycophagists (Elliott et al. 2022). Dispersed fungal propagules could include spores or vegetative tissue, such as hyphal fragments and fragments of mycelial structures in colonised roots (Vašutová et al. 2019), although the viability of these propagules through gut passage was not specifically tested in this study. Their inferred dispersal of ectomycorrhizal fungi plays a vital role in ecosystems by enhancing plant nutrient uptake, facilitating carbon cycling, and boosting overall productivity (Courty et al. 2010). Ectomycorrhizal fungi are also capable of enhancing plant growth and conferring resistance to pathogens and drought by expanding plant root access to soil resources (Azcón‐Aguilar and Barea 1997; Ruiz‐Lozano 2003). Conversely, reduced ectomycorrhizal colonisation has been associated with severe ecological disruption (Hopkins et al. 2018; Sapsford et al. 2021). We similarly infer that the consumption of saprotrophic fungi by both bettong species represents their role in critical nutrient cycling and decomposition processes. Saprotrophic as well as pathogenic fungi contribute significantly to the breakdown and recycling of organic matter, ensuring the conversion of energy and nutrient acquisition from dead or decaying plant and animal tissue across ecosystems (Talbot et al. 2013). Future research investigating the viability of fungal propagules through the gut passage of boodies and woylies would strengthen inference on dispersal capabilities of these species.

The comparable consumption of ectomycorrhizal and saprotrophic fungi by boodies and woylies suggests potential functional redundancy in fungal dispersal where both species co‐occur. Additionally, both species have been shown to be dispersers of viable seeds (Beca et al. 2020; Palmer, Valentine, et al. 2021) and are able to improve soil conditions, including enhancing soil moisture and nutrient levels, while reducing compaction through their digging behaviour (Beca 2021; Palmer, Valentine, et al. 2021). However, the semi‐fossorial nature of boodies sets them apart from woylies, where their warrens create microsites that provide habitat for a range of native mammal and reptile communities (Beca et al. 2021; Palmer, Valentine, et al. 2021).

## 5 Conclusions and Conservation Implications

Our study provides detailed insights into the mycophagous diets of boodies and woylies, revealing that despite considerable diversity in study sites and environmental gradients, both species consume a high abundance of fungi from the family Pezizaceae, particularly of the truffle‐like genus *Ruhlandiella*. While both bettongs provide important ecosystem functions and may exhibit some functional redundancy at broad scales, minute differences in secondary fungal resource utilisation may enhance dispersal and proliferation of diverse fungal taxa across the landscape. Additionally, boodies offer unique ecosystem engineering benefits through burrow construction that creates habitats for other native mammals and reptiles (Palmer, Valentine, et al. 2021), highlighting the complementary conservation value of establishing both species in restoration programs. Given the comparable aridity index values between Dirk Hartog Island and Faure Island, the target translocation site has the potential to support similar fungal community composition and diversity, suggesting favourable conditions for mycophagous mammal establishment. Importantly, our study draws comparable inferences from predominantly closed landscapes (fenced reserved and islands) to inform translocation decisions to another closed landscape (Dirk Hartog Island). However, in planning for future translocations to unrestricted systems, research incorporating open landscape populations would be valuable for addressing broader questions about mycophagous mammal ecology.

A concern that becomes particularly relevant when considering these sympatric translocations is the dietary similarities between these species, which have the potential to result in competition for resources during periods of drought and population irruption (Bice and Moseby 2008; Hayward et al. 2010; Moseby et al. 2018; Short 2022; Treloar et al. 2021). Here, we recommend undertaking a comprehensive resource assessment of the release site prior to translocations of bettongs, alongside careful consideration of carrying capacity in order to make management decisions to minimise interspecific competition. Translocations should also be planned to coincide with post‐rainfall periods when fungal fruiting is optimal (Trappe et al. 2008), and should incorporate extended post‐release monitoring that encompasses dietary acclimatisation phases and, particularly in arid environments, spans complete drought cycles to ensure long‐term establishment success (Bannister et al. 2021).

## Author Contributions

**Rebecca J. Quah:** conceptualization (equal), data curation (lead), formal analysis (lead), funding acquisition (lead), investigation (lead), methodology (equal), project administration (lead), resources (equal), visualisation (lead), writing – original draft (lead), writing – review and editing (equal). **Saul J. Cowen:** conceptualization (equal), methodology (equal), supervision (equal), writing – review and editing (equal). **Robert A. Davis:** conceptualization (equal), methodology (equal), supervision (equal), writing – review and editing (equal). **Harriet R. Mills:** conceptualization (equal), methodology (equal), supervision (equal), writing – review and editing (equal). **Anna J. M. Hopkins:** conceptualization (equal), methodology (equal), supervision (lead), validation (lead), writing – review and editing (equal).

## Funding

This work was supported by the Australian Wildlife Society's University Research Grant and Edith Cowan University's Higher Degree by Research funds.

## Ethics Statement

Samples used in this study were collected under DPIRD animal ethics 23–03‐19 (Faure Island and Mt. Gibson); DBCA animal ethics 2021‐56C/57D (Matuwa); UWA animal ethics RA/3/100/1667 (Dryandra); and DAF animal ethics CA 2024/03/1839 (Newhaven).

## Conflicts of Interest

The authors declare no conflicts of interest.

## Data Availability

The dataset of raw molecular sequences generated and analysed during the current study is available in the Sequence Read Archive (SRA) curated by NCBI under the accession number PRJNA1360078.
